# Effects of control temperature, ablation time, and background tissue in radiofrequency ablation of osteoid osteoma: A computer modeling study

**DOI:** 10.1002/cnm.3512

**Published:** 2021-08-08

**Authors:** Ricardo Rivas, Rudy B. Hijlkema, Ludo J. Cornelissen, Thomas C. Kwee, Paul C. Jutte, Peter M. A. van Ooijen

**Affiliations:** ^1^ Department of Radiotherapy University of Groningen, University Medical Center Groningen Groningen The Netherlands; ^2^ Faculty of Mathematics and Natural Sciences University of Groningen Groningen The Netherlands; ^3^ Department of Radiology University of Groningen, University Medical Center Groningen Groningen The Netherlands; ^4^ Department of Orthopedics University of Groningen, University Medical Center Groningen Groningen The Netherlands

**Keywords:** computer models, finite element method, osteoid osteoma, radiofrequency ablation

## Abstract

To study the effects of the control temperature, ablation time, and the background tissue surrounding the tumor on the size of the ablation zone on radiofrequency ablation (RFA) of osteoid osteoma (OO). Finite element models of non‐cooled temperature‐controlled RFA of typical OOs were developed to determine the resulting ablation radius at control temperatures of 70, 80, and 90°C. Three different geometries were used, mimicking common cases of OO. The ablation radius was obtained by using the Arrhenius equation to determine cell viability. Ablation radii were larger for higher temperatures and also increased with time. All geometries and control temperatures tested had ablation radii larger than the tumor. The ablation radius developed rapidly in the first few minutes for all geometries and control temperatures tested, developing slowly towards the end of the ablation. Resistive heating and the temperature distribution showed differences depending on background tissue properties, resulting in differences in the ablation radius on each geometry. The ablation radius has a clear dependency not only on the properties of the tumor but also on the background tissue. Lower background tissue's electrical conductivity and blood perfusion rates seem to result in larger ablation zones. The differences observed between the different geometries suggest the need for patient‐specific planning, as the anatomical variations could cause significantly different outcomes where models like the one here presented could help to guarantee safe and successful tumor ablations.

## Graphical abstract

How does the background tissue affect the outcome of radiofrequency ablation of osteoid osteoma for typical configurations and treatment protocols? To study their effects, computer simulations using the finite element method for three different geometries and control temperatures of 70, 80, and 90°C were used to obtain the resulting ablation radius. The ablation radius showed a dependency not only on the properties of the tumor but also on the background tissue.

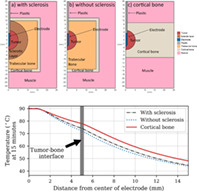

## 1 INTRODUCTION

Radiofrequency ablation (RFA) is a minimally invasive technique that has become the treatment of choice for osteoid osteomas (OO), one of the most common types of bone tumors.^1^ Since Rosenthal et al's initial findings,^2^ different settings have been tested to guarantee optimal treatment. Currently, the standard RFA for OO is a temperature‐controlled mode of 90°C for 6 min,^1^ which originates from the first Rosenthal et al's experiments. Originally, they had decided on an ablation time of 3 min with a control temperature of 90°C, but the duration was later increased to 6 min after a (successful) attempt to reduce the number of tumor recurrences in.^2^, ^3^ By increasing the ablation time, the amount of RF energy delivered to the tissue is increased, causing larger ablation zones.

Even though the ablation time is usually between 4 and 6 min, various studies have used longer times, ranging generally from 6 to 10 min.^4^, ^5^, ^6^, ^7^, ^8^, ^9^, ^10^ In the case of electrodes of a smaller diameter, which produce smaller ablation volumes,^11^ ablation times of up to 14–15 min of active heating have been used^12^ in an attempt to compensate for their reduced ablation volumes. However, the differences in ablation size between the standard approach of approximately 6 min and what others have proposed has not been quantified, and only the clinical outcomes were presented. Although the series are mostly successful, it is not clear what the ideal ablation time is, and what the quantitative effects of changing it are. Given that the main aim of the treatment is a reduction in pain, questionaries to assess the severity of the patient's pain are the main metric utilized before and after treating OO with RFA. Thus, post‐operative imaging studies are not usually performed, making it hard to quantify the effects of changes in protocol on the ablation zone.

Goldberg et al studied RFA on ex‐vivo liver and muscle tissues and showed that the resulting ablation radius did not change after 6 min^11^ had passed, which coincides with the initial suggestions by Rosenthal et al. However, the conclusions of this and other similar studies of RFA on liver or other soft tissues may not apply to bony tissues, hence the need for a systematic study of the effects of ablation time on OO.

Other clinical studies also opted to vary the control temperature, with most of them ranging from 60 to 90°C.^7^, ^8^, ^9^, ^13^, ^14^ Generally, lower temperatures are used to prevent damaging nearby structures at risk like nerves (as this is expected to lead to a reduced ablation zone), and sometimes it may also be difficult to actually attain the target of 90°C and thus lower temperatures are used. However, again, the lack of post‐operative imaging makes it hard to understand the effects on the thermal damage caused by the changes in the used control temperature.

To the best of our knowledge, no systematic study has been done on the consequences of varying the control temperature and ablation time of RFA on OO. These parameters have a direct impact on the extension of the ablation zone and therefore on the clinical outcome but testing on patients carries the risk of unsuccessful ablations, which could be detrimental to the patient's health. Therefore, we implemented computer models using the finite element method (following the previous work on OO by Irastorza et al^15^) to study the effect of control temperature and ablation time on ablation size. We tested this on three typical anatomical configurations of OO and looked at how the different tissues surrounding the tumor affected the extension of the ablation.

## 2 MATERIALS AND METHODS

Finite element models of non‐cooled temperature‐controlled RFA of typical OOs were developed, following mostly the work by Berjano^16^ and Irastorza et al,^15^ to determine their dynamic temperature distribution and cell death at various control temperatures and durations. Based on the studies mentioned in the introduction, the models were studied with control temperatures of 70, 80, and 90°C, and for as long as 15 min of active heating.

### 2.1 Description of the geometry

**FIGURE 1:**
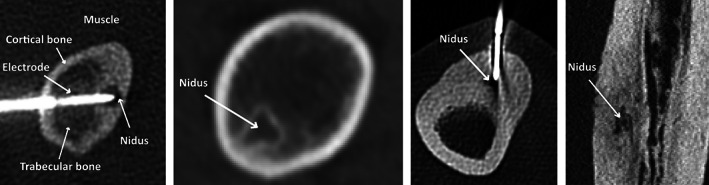
Three examples of common cases of osteoid osteoma (OO). The hypodense focus corresponds to the location of the nidus. The first image shows a nidus with a thin layer of sclerosis surrounded by both cortical and trabecular bone in a femur. The second image shows a nidus with little sclerosis and surrounded mostly by trabecular bone. The third image shows an intracortical nidus completely covered by cortical bone in a tibia. The right‐most image shows another intracortical OO but now from a different view. The first and third images show an example of how the electrode (the hyperdense‐bright object) is usually placed in the nidus. The first image is labeled to work as a reference, and in the following ones the tumor was indicated for clarity

**FIGURE 2:**
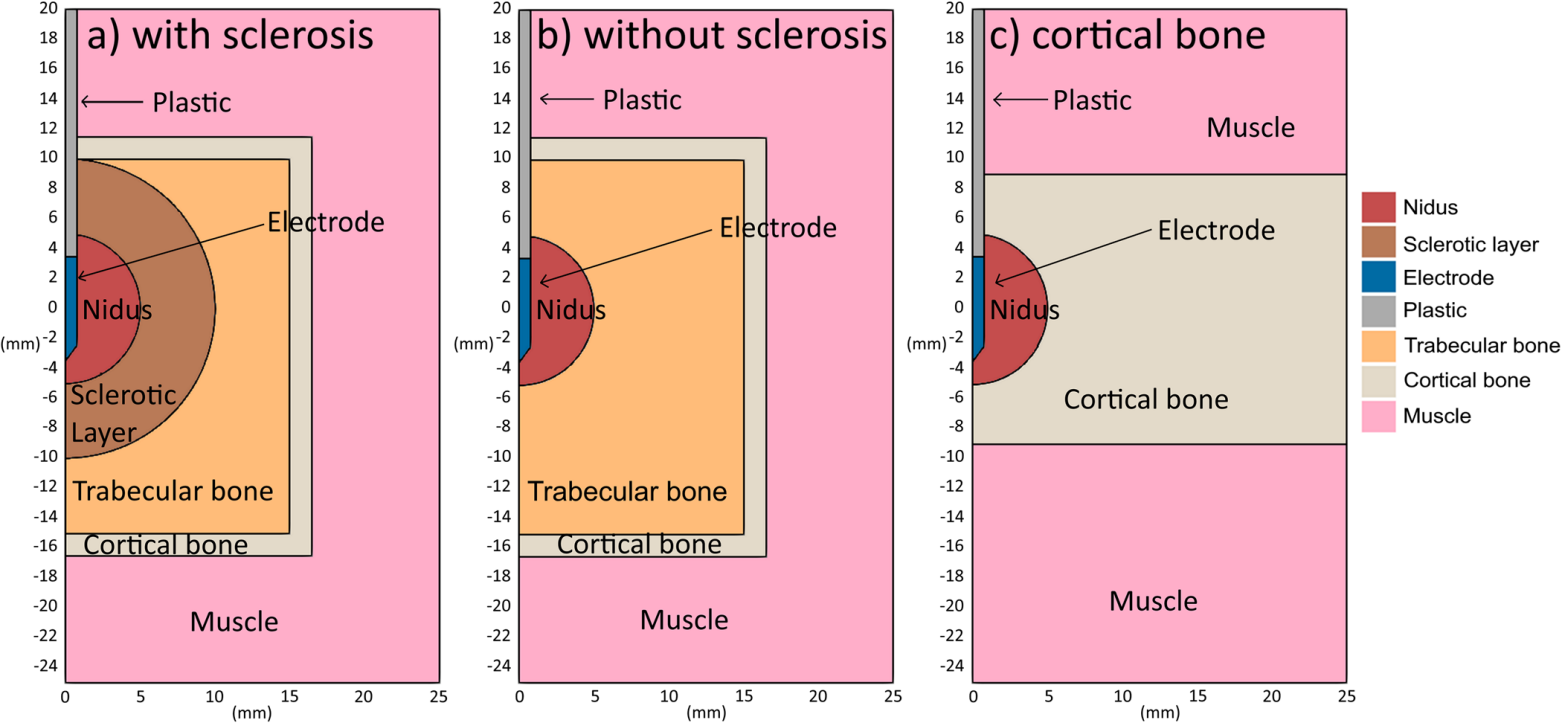
Geometries mimicking typical cases of osteoid osteoma (OO), roughly matching the cases from Figure 1. (A) is a case of an OO surrounded by a layer of sclerosis. (B) is a case of an OO in the middle of trabecular bone close to a cortical wall. (C) is a case of an OO completely surrounded by cortical bone, and that could also represent the extent of thermal ablation along the longitudinal axis of bone. All three cases are further surrounded by a layer of muscle

There were two main objectives when creating the geometries: (1) to approximate common cases of OO and (2) to explore the effects of different anatomical configurations of RFA on OO. Examples of common cases can be seen in Figure 1. With these cases in mind, we decided to test three different scenarios: The first, a juxtacortical nidus (the tumor) surrounded by a layer of sclerosis which in turn is surrounded by trabecular and cortical bone (Figure 2A). The second, similar to the first, but without the sclerotic layer and with the nidus surrounded by trabecular bone (Figure 2B). The third, an intracortical nidus with no trabecular bone or sclerotic layer directly around it (Figure 2C). The third case could also serve as an example of how the ablation radius changes in the longitudinal direction of bone. All the geometries had a final layer of muscle surrounding the cortical bone. The nidus, a sphere with a 1 cm diameter, was treated with an electrode with an active tip of 0.75 cm in length and gauge 17, in accordance with common practice by interventionists. To simplify the complex anatomy, a rectangular cortical bone shape was chosen with dimensions similar to those of a typical tibia. All the geometries were defined as 2D axisymmetric, with the axis of symmetry along the electrode. Given that most of the cell death occurs close to the electrode, we found these geometrical approximations to be sufficient for the tests; increasing the distance further or changing the shape of the cortical box or the surrounding muscle did not change the outcome significantly. Mesh convergence tests were performed on the three models to guarantee mesh independence in our results, and special attention was paid to the size of the elements next to the electrode where the highest gradients were located. The cell death radius (explained in the next section) was used as the convergence criterion. Convergence was assumed to have occurred when a change of less than 1% in the cell death radius was obtained. Triangular elements with a characteristic length as small as .1 mm were tested.

### 2.2 Equations governing radiofrequency ablation

In RFA, heat is generated due to Joule heating which then propagates through the body. The simulations thus consisted of solving a coupled electric‐thermal problem, in which the thermal problem was governed by Pennes' bioheat equation^17^ modified to account for phase change due to tissue vaporization^18^:

(1) $$\frac{\partial h}{\mathrm{\partial t}}=\nabla \cdot \left(k_{i}\left(T\right)\nabla T\right)+Q_{\mathrm{RF}}-Q_{p}$$

where *h* is the enthalpy, and $k_{i}$ is the thermal conductivity (w/m∙K). These properties are temperature dependent, as described by the piece‐wise functions below. *T* is the temperature (K), *t* time (s) and $Q_{\mathrm{RF}}$ the heat source ($W/m^{3}$). $Q_{p}$ is the blood perfusion heat loss ($W/m^{3}$) and is defined as:

(2) $$Q_{p}=\omega_{i}\left(\Omega \right)\rho_{b}c_{b}\left[T-T_{b}\right]$$

where $\rho_{b}$ is the density of blood ($\mathrm{kg}/m^{3}$), $c_{b}$ the specific heat of blood (J/kg∙K), $T_{b}$ the temperature of blood (K) $.\omega_{i}$ is the tissue‐dependent perfusion coefficient ($s^{-1}$) as a function of the cell viability Ω. The perfusion of each cell is kept constant and becomes zero when it dies, as specified by the cell death model.

A quasi‐static approach was used for the electrical problem as it is known that tissues in the area of interest can be seen as totally resistive to the RF ablation frequencies (≈500 kHz).^19^ Then, the heat source $Q_{\mathrm{RF}}$ is given by:

(3) $$Q_{\mathrm{RF}}=\sigma_{i}{\left|E\right|}^{2}$$

where *E* is the electric field intensity (V/m) and $\sigma_{i}$ is the electrical conductivity (S/m) of each tissue. The electric problem is then stated by the Laplace equation:

(4) $$\nabla \cdot \sigma_{i}\left(T\right)\nabla V=0$$

where *V* is the root mean squared value of the applied voltage (V).

The following boundary conditions were set: a Neumann boundary condition of zero thermal flux at the symmetry axis and a Dirichlet boundary condition of constant temperature of 37°C at the outer boundaries for the thermal problem. Also, a Dirichlet boundary condition of zero voltage at the outer boundaries to mimic the grounding pad, a Neumann boundary condition of zero flux at the symmetry axis, and a Dirichlet boundary condition with a value equal to the applied r.m.s. voltage at the electrode surface were employed for the electric problem.

To mimic the clinical setting, the voltage was regulated such that a pre‐defined temperature at the tip of the electrode was reached, and subsequently maintained, which can be modeled by using a PI‐controller, i.e.

(5) $$V_{r.m.s.}=K_{p}\left(T_{\text{target}}-T\left(t\right)\right)+K_{i}\int_{0}^{\tau }\left(T_{ta\text{rget}}-T\left(t\right)\right)\;\mathrm{dt}$$

where $V_{r.m.s.}$ is the r.m.s. voltage (V) set at the electrode boundary, $T_{\text{target}}$ the desired control temperature, $T\left(t\right)$ the temperature at the tip at time *t*, $K_{p}$ the error proportionality constant, and $K_{i}$ the integral proportionality constant. The initial voltage was set to zero. Then the $K_{p}$ and $K_{i}$ values had to be found with similar behaviors as in the ex‐ Bitsch et al experiments.^20^ Although this is model dependent, a good agreement was found by setting $K_{p}$ at 1.15 V/K and $K_{i}$ at 0.06 V/K/s.^21^

For most of the model, the Trujillo and Berjano^22^ model propositions for biological tissues, temperature‐dependent parameters of electrical conductivity, thermal conductivity, and apparent heat capacity were used as reference.

Regarding electrical conductivity, a linear increase of 1.5% per degree Celsius until the point of vaporization was used. Then, vaporization is characterized as a sudden drop in the electrical conductivity, with a linear decrease up to a factor of 10,000, as described in Equation (6).

(6) $$\sigma_{i}\left(T\right)=\left\{\begin{array}{c} \begin{array}{cc} \sigma_{i}+∆\sigma_{i}\left(T-37{}^{\circ}C\right),\; & \;T\leq 100{}^{\circ}C \end{array}\\ \begin{array}{cc} \sigma_{100{}^{\circ}C}+\left(\sigma_{\mathrm{vap}}-\sigma_{100{}^{\circ}C}\right)\frac{\left(T-100{}^{\circ}C\right)}{5}, & \;100{}^{\circ}C<T\leq 105{}^{\circ}C \end{array}\\ \begin{array}{cc} \sigma_{\mathrm{vap}}, & \;T>105{}^{\circ}C \end{array} \end{array}\right.$$

where $\sigma_{i}$ is the baseline electrical conductivity (S/m) of each tissue at 37 °C, ∆*σ* is the 1.5% temperature dependent rate of change in electrical conductivity per degree Celsius, and $\sigma_{\mathrm{vap}}$ is the modeled electrical conductivity of vaporized tissue where $\sigma_{\mathrm{vap}}=10x10^{-3}$ (S/m).^22^

The thermal conductivity was considered as having a linear increase until the point of vaporization where a maximum value was set for any temperature beyond 100 °C:

(7) $$k_{i}\left(T\right)=\begin{array}{c} \left\{\begin{array}{c} \begin{array}{cc} k_{i}+∆k_{i}\left(T-37{}^{\circ}C\right),\; & \;T\leq 100{}^{\circ}C \end{array}\\ k_{i}+∆k_{i}\left(100{}^{\circ}C-37{}^{\circ}C\right)\begin{array}{cc} , & \;T>100{}^{\circ}C \end{array} \end{array}\right. \end{array}$$

where $k_{i}$ is the baseline thermal conductivity of each tissue at 37°C and $∆k_{i}$ corresponds to a 0.003 change in thermal conductivity per degree Celsius.^15^, ^23^

When water starts to evaporate there is a sudden change in the heat capacity during the phase‐change. Although tissue vaporization is not expected because the PI controller will regulate the output current to maintain a maximum target temperature of 90 °C, tissue vaporization was modeled using the enthalpy method^18^, ^22^:

(8) $$h=\begin{array}{c} \left\{\begin{array}{c} \begin{array}{cc} \rho_{i}c_{i}\left(T-37{}^{\circ}C\right),\; & \;37{}^{\circ}C\leq \;T\leq 99{}^{\circ}C \end{array}\\ \begin{array}{cc} h\left(99\right)+h_{\mathrm{fg}}C_{i}\;\frac{\left(T-99{}^{\circ}C\right)}{\left(100{}^{\circ}C-99{}^{\circ}C\right)}, & \;99{}^{\circ}C<T\leq 100{}^{\circ}C \end{array}\\ h\left(100\right)+\rho_{\mathrm{vap}}c_{\mathrm{vap}}\left(T-100{}^{\circ}C\right)\begin{array}{cc} , & \;T>100{}^{\circ}C \end{array} \end{array}\right. \end{array}$$

where $\;\rho_{i}c_{i}$ represent the baseline density $\left(\frac{\mathrm{kg}}{m^{3}}\right)$ and specific heat $\left(\frac{J}{\mathrm{kgK}}\right)$ of each tissue, $\rho_{\mathrm{vap}}$(370 $\frac{\mathrm{kg}}{m^{3}}$) and $c_{\mathrm{vap}}$ (2156 $\frac{J}{\mathrm{kgK}})$ are the density and specific heat of vaporized tissue,^22^ $h_{\mathrm{fg}}$ is the latent heat of vaporization (2.25 x $10^{6}\frac{J}{\mathrm{kg}}$) and $C_{i}$ is the water fraction of each tissue.

FEniCS,^24^ an open‐source platform for solving partial differential equations, was used to develop a solver for our models. The implementation was based on the works by Hall,^25^ who developed a model to solve various minimally invasive tumor ablation therapies (e.g., RFA, Microwave ablation, etc.) using FEniCS.

### 2.3 Tissue properties

Most of the tissue properties were obtained from,^26^ except for the sclerotic layer and nidus (where we followed Irastorza's assumptions), or as indicated otherwise in Table 1. We also followed Irastorza's assumed blood perfusion values for all tissues. The properties of plastic and metal were taken from.^27^ In most cases, where a range of values was available, the average value was chosen. The only exceptions where in the specific heat and thermal conductivity of the reactive zone, for which we used cortical bone values to simulate the effects of a high degree of sclerosis but still with some blood perfusion.

**TABLE 1:** Material's properties

Material/tissue	Density (kg/m^3^)	Electrical conductivity (S/m)	Specific heat (J/kg∙K)	Thermal conductivity (W/m/K)	Blood perfusion coefficient (x $\;10^{-4}s^{-1})$	Water fraction
Electrode	6450^a^	1.00E+08^a^	840^a^	18^a^	0	0
Plastic	70^a^	1.00E‐05^a^	1045^a^	0.026^a^	0	0
Nidus	1046^b^	0.22^b^	2726^b^	0.56^b^	48^b^	0.60 ^d^
Sclerotic layer	1908^b^	0.0535^b^	1313*	0.32*	2.95^b^	0.23 ^c^
Trabecular bone	1178	0.0867	2274	0.31	5.90^b^	0.27 ^c^
Cortical bone	1908	0.022	1313	0.32	0.00^b^	0.23 ^c^
Muscle	1090	0.446	3421	0.49	6.70^b^	0.76 ^e^

*Note*: All properties were obtained from Reference 26 for tissues at 500 kHz, except where marked. The references correspond to a, Reference 27; b, Reference 15; c, Reference 28; d, Reference 29; e, Reference 30.

^*^ Correspond to the values of cortical bone from Reference 26. The water fraction of connective tissue was assumed for the nidus and the water fraction of cortical bone was assumed for the sclerotic layer.

### 2.4 Cell death model

To assess the tissue damage caused by the heating process we used the Arrhenius damage model.^31^ The cell viability of a given tissue is given by Ω, which calculates the thermal damage to each cell over time:

(9) $$\Omega \left(t\right)=\int_{0}^{t}{\mathrm{Ae}}^{-\frac{∆E}{\mathrm{RT}\left(\tau \right)}}\;d\tau ,$$

where *R* is the universal gas constant, *A* (a frequency factor), and ∆E (the activation energy for the irreversible damage reaction) the cell‐line dependent parameters. Since the treatment is on bone tumors, and Tillotson et al found that cortical and trabecular bone have roughly the same susceptibility to heat,^32^ osteocytes seemed like the best option from the cell‐line parameters found in literature, with values of $A=8.99\;x\;10^{133}s^{-1}$ and ∆E=838kJ/mol.^33^

Cell death (Ω) is given as a probability of cell viability, where exposure to higher temperatures for longer periods increases the likelihood of a given cell dying. A value of Ω = 4.6 was chosen as the threshold, corresponding to a 99% probability of cell death. Once a cell reached said threshold, it was considered dead and the perfusion was stopped. To calculate the outcome for our studies, a radius from the center of the electrode in directions perpendicular and parallel (upwards) to the electrode was used to calculate the resulting cell death radius in both directions. Additionally, we allowed the simulations to cool down for 5 min (chosen arbitrarily based on preliminary results) after the active electrode was turned‐off to capture the thermal damage more accurately over time while the heat was still dissipating, as shown in Reference 33.

### 2.5 Validation of the model

Our model is based on the computer model by Irastorza et al,^21^ who modeled RFA of OO and compared their results to the ex‐vivo experiments by Bitsch et al.^21^ To validate our model, the Irastorza et al model was replicated as best as possible and the results from our model were compared against their results and against the ex‐vivo experiment by Bitsch et al. The values from their studies were obtained with WebPlotDigitizer^34^ and compared against the results from our simulations.

Bitsch et al created three OO models using bovine long bone specimens, categorized depending on the thickness of the cortical bone lamella separating the nidus from the periosteum. A hole was drilled and filled with 0.8% agarose gel to model the nidus. In the soft tissue surrounding bone, three thermocouples were placed at distances of 0, 5, and 10 mm from the periosteum. The specimens were heated to an internal temperature of 35°C, and RFA was performed for 400 s with a control temperature of 95°C. The temperatures measured by the thermocouples were reported in graphs. The ex‐vivo setting was then replicated by Irastorza et al using the temperature profiles obtained from the temperature probes to validate and optimize their OO model. Of the three Bitsch et al models, the one with a 3 mm lamella thickness was chosen arbitrarily here, but our model was compared against all their configurations. For a more detailed explanation of the ex‐vivo experiments and the assumptions to replicate them, please refer to Bitsch et al^20^ and Irastorza et al,^21^ respectively.

## 3 RESULTS

### 3.1 Validation

**FIGURE 3:**
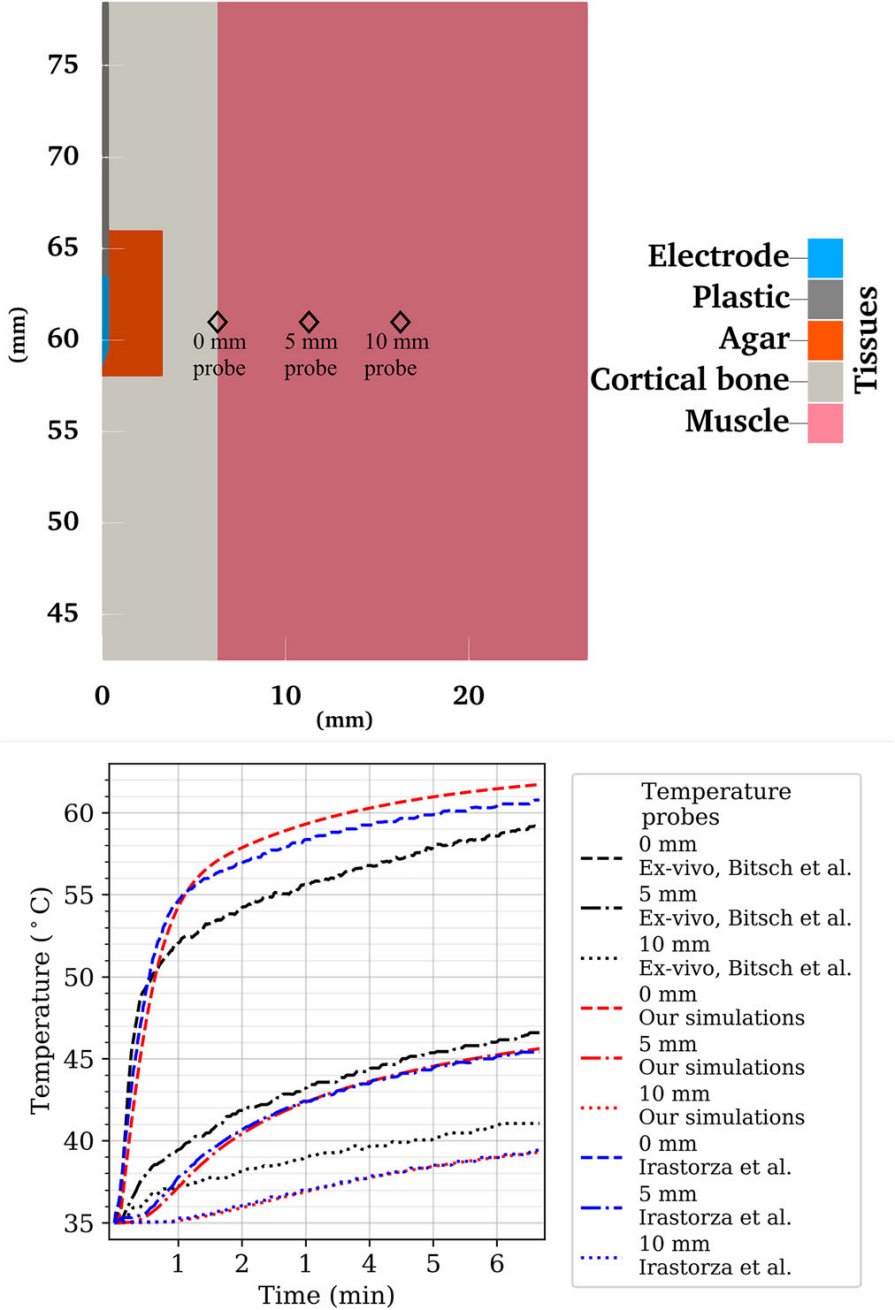
Top: Geometry used to replicate one of the Bitsch et al ex vivo experiments, where temperature probes were positioned at 0‐, 5‐, and 10‐mm distance from the lamella, here shown as the black rhombuses in the geometry. Bottom: Temperature in time for the three probes. In black, Bitsch et al ex vivo experiments; in red, our simulations; in blue, Irastorza et al's simulations

The results were within 1°C degree of difference to the ones obtained by Irastorza et al for most of the ablation time with a maximum absolute error of 2.2°C, encountering the largest differences for the probe closes to the tumor and almost identical results for the other two. Against the Bitsch et al results, a maximum absolute error of 4.3°C of difference was found. Figure 3 shows the geometry of the model and the resulting temperature through time from the virtual and ex‐vivo probes at 0, 5, and 10 mm from the lamella.

### 3.2 Effects of time and control temperature

**FIGURE 4:**
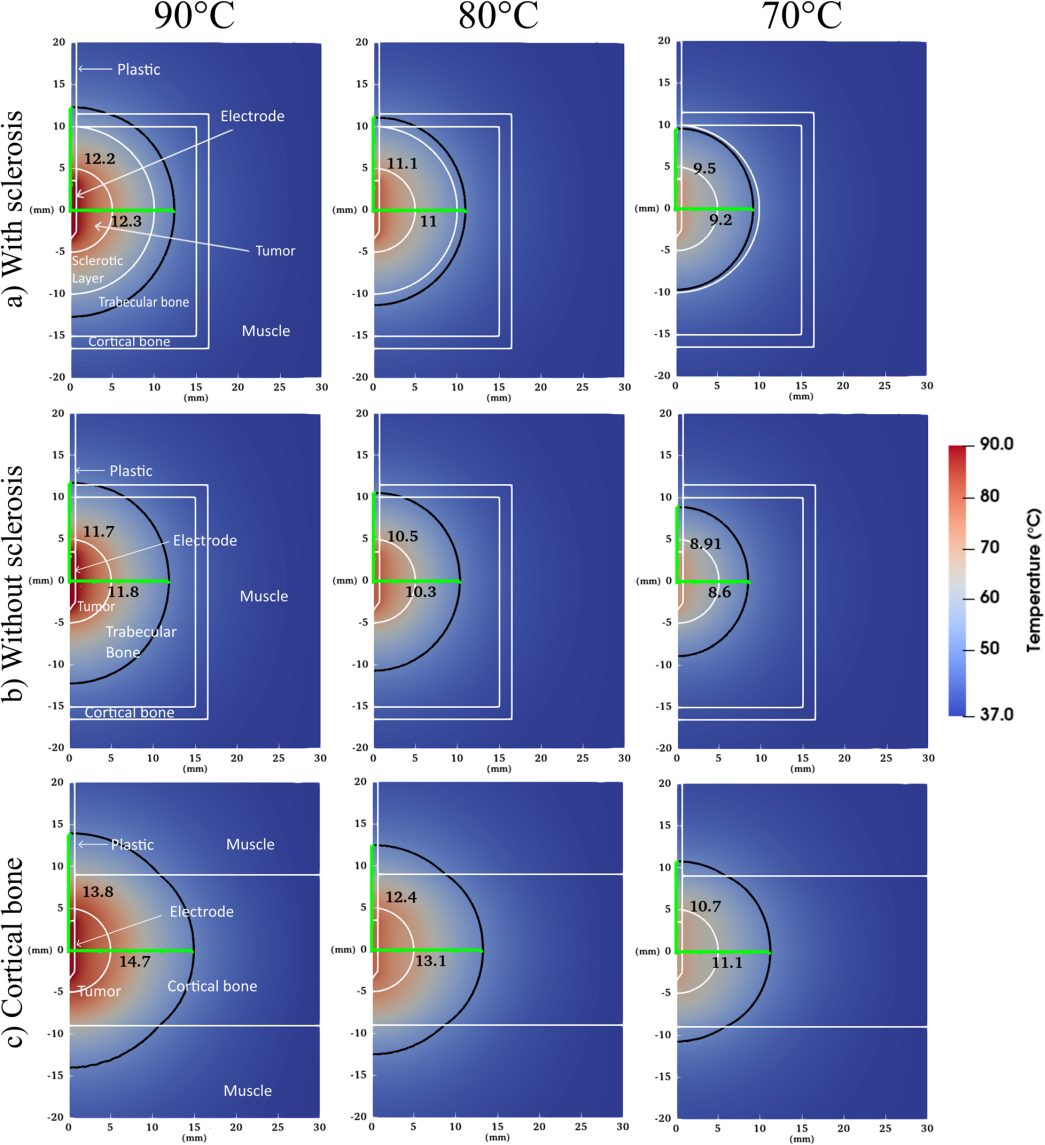
Simulated temperature distribution for the three different geometries and three control temperatures after 15 min of ablation. White lines represent the geometry of the model and the black line represents the resulting cell death isoline. The green line indicates the distance from the center of the electrode to the isoline, that is, the radius used as the outcome variable

Figure 4 shows the temperature distributions of the three tested simulation models at the end of active heating (at 15 min), for all control temperatures. Cell death and measurements are also shown, obtained after 15 min of ablation +5 min of cooldown. None of the tested models and configurations tested reached maximum temperatures of ≥100**°**C, and there was no tissue vaporization.

**FIGURE 5:**
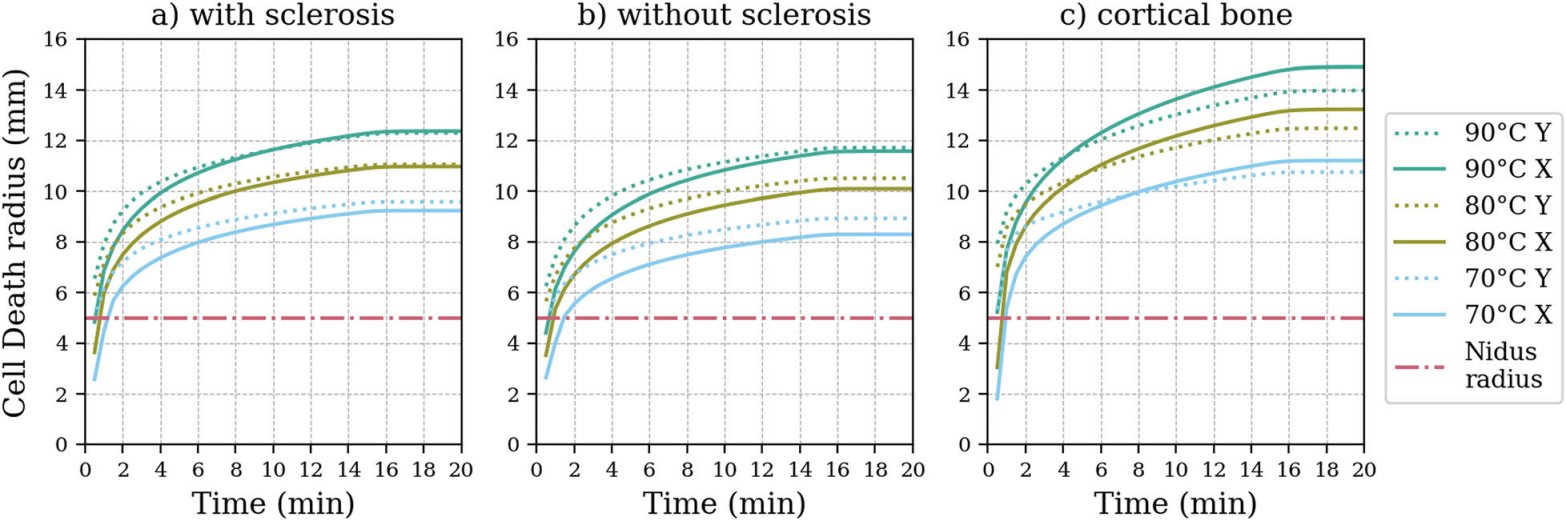
Radii of the cell death isoline obtained for the geometries tested under all the configurations. The number in the labels indicates the control temperature and the *X* or *Y* indicate whether it was measured in direction perpendicular (*X*) or parallel (*Y*) to the electrode. The straight dash‐dot line indicates the radius of the nidus. The first few initial values were omitted for clarity

Figure 5 shows all the radii obtained for all the geometries tested with different control temperature configurations and durations. The dash‐dot line corresponds to the radius of the nidus, to enable visual comparison of the obtained radius in each configuration against the target tissue to be ablated (the nidus). The number in each label indicates the control temperature used. The *X* and *Y* indicate the directions in which the temperature isotherm was obtained: *X*, perpendicular to the electrode; *Y*, parallel to the electrode. Both measurements start at the center of the electrode, and the *Y* direction was measured from the center to the top.

The ablation radii were larger at higher temperatures, and also increased with time, as expected. The ablation radii of all the tested geometries and control temperatures were larger than the tumor at the end of the ablation. The ablation radius developed rapidly in the first few minutes and then slowed down by the end of the ablation. The ablation radius was smallest for geometry (b), followed by geometry (a), and largest in geometry (c), when compared at the same control temperature. The radius was almost identical in both directions for the first two cases, whereas in geometry (c), the ablation radius increased considerably more in the direction perpendicular to the electrode after a couple of minutes for all the tested control temperatures.

**FIGURE 6:**
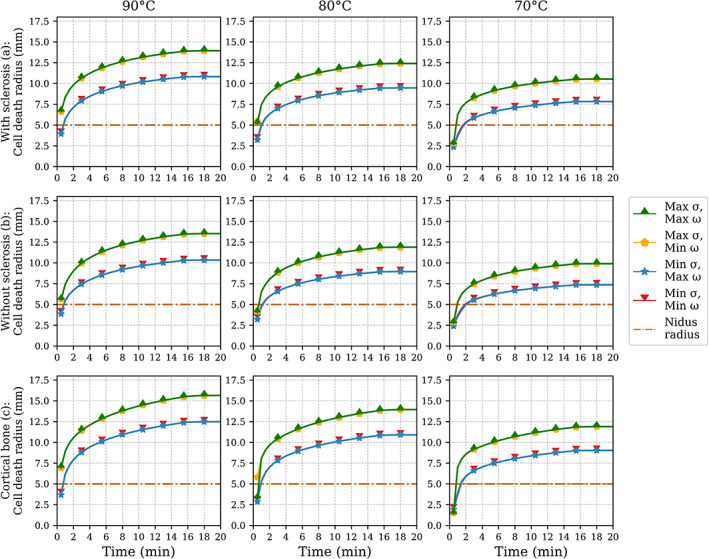
Analysis on the effects of the tumor's electrical conductivity (*σ*) and blood perfusion (*ω*) on the resulting maximum ablation. Combinations of maximum and minimum values of *σ* and *ω* were tested in all three geometries, as described in the labels. The straight dash‐dot line indicates the radius of the nidus. The initial first few initial values were omitted for clarity

To test the sensitivity of the models further to changes in the tissue parameters, we performed simulations with four combinations of maximum and minimum electrical conductivity (0.5 and 0.08 S/m, respectively) and blood perfusion (70 x $10^{-4}s^{-1}$ and 26 x $10^{-4}s^{-1}$) values in the nidus. These parameters were chosen because they are the most influential factors defining the size of the ablation radius.^35^ The values were taken from Irastorza et al's study.^15^ For all target temperatures and anatomical configurations, the ablations were performed for 15 min of active heating plus 5 min of cooldown at the end. Only the material parameters of the tumor were changed because the three geometries tested already presented great variations in the tissues surrounding the tumor. The results in Figure 6 show that the ablation radius changes depended mostly on the effects of electrical conductivity, with the perfusion having little to no effect. Compared to the ablation radius with the baseline parameters, a change in the ablation zone radius of approximately +15% for the cases with a higher nidus electrical conductivity and −15% for the cases with the lower electrical conductivity was seen for all configurations. Additionally, since the parallel and perpendicular radii showed the same kind of relative relationship between them as the average cases already explored, we omitted the parallel radius from the graphs for clarity purposes.

**FIGURE 7:**
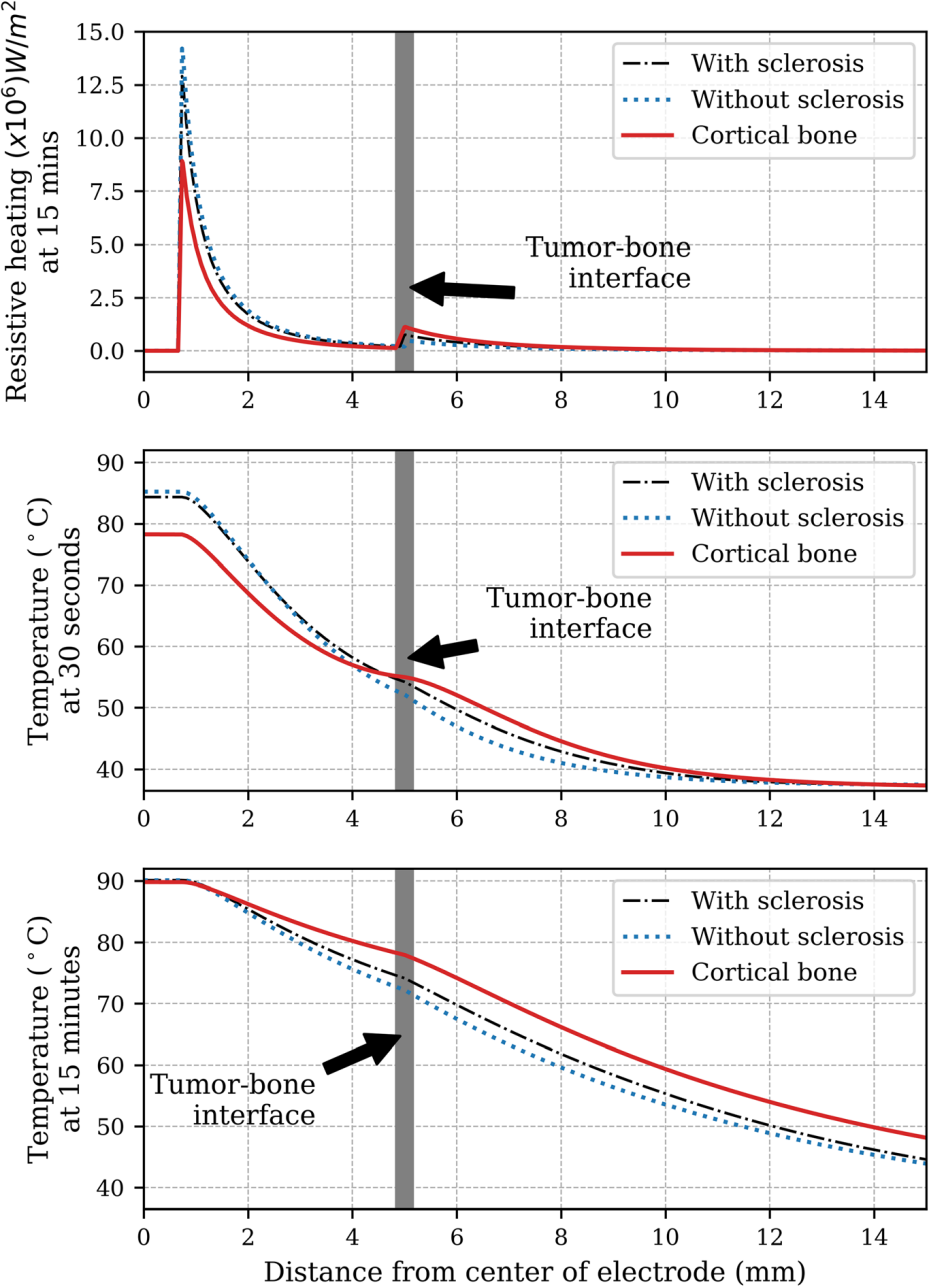
Line graphs of the resistive heating and temperature of the three models tested, from the center of the electrode and perpendicular to it, with a control temperature of 90°C. The tissue interface between the tumor and bone is located at 5 mm. Two representative points in time were chosen for the temperature profiles, one close to the beginning of the procedure (30 s) and one at the end (15 min) to highlight the contrast in the distribution of heat at the beginning and at the end of the ablation. The distribution of the resistive heating did not change significantly over time, so only one line graph is shown, obtained at 15 min

Finally, to understand the reason for the differences in the ablation radii in the different models better, we obtained a line graph of the resistive heating and the temperature from the center of the electrode and perpendicular to it. Only cases with a control temperature of 90°C are presented as examples but the trends presented in Figure 7 were true for all the control temperature configurations.

The distribution of the resistive heating demonstrates clear differences in the distribution of the induced heat that depend did not only depend on the target tissue (nidus) but on the surrounding tissue as well. This is particularly clear in the peak at the electrode‐tumor interface (at 0.75 mm) and at the tumor‐bone interface (at 5 mm). The amount of induced heat varied with time as a function of the voltage applied but its distribution remained more or less the same all the time. Hence, only one graph of the 15 min measurement is shown.

The temperature profile also shows the effects of the differences on the distribution of induced heat. At the beginning of the ablation, the case with the tumor surrounded by cortical bone seems to induce less heat around the tumor, but creates an additional peak of heat at the tumor‐bone interface, which, with time, increases the maximum temperatures reached deeper in the tissue. It is also interesting to note that all the cases seem to have similar heat transfer rates after the tumor (at 5 mm), as shown in the slope of the temperature profile.

## 4 DISCUSSION

RFA is a technique that has been consolidated as a standard clinical practice for the treatment of OO due to its effectiveness and low complication rates. However, neither the effects of the ablation time, nor the control temperature, nor the effects of the background tissue surrounding the tumor on the resulting ablation zone are well understood. Some studies have used ablation times as short as 4 min^36^ whereas in others the time has been as long as 15 min^12^ to treat similar types of lesions. Also, deliberate variations in control temperature, with the aim of generating smaller ablation zones, have been reported, usually for small tumors or tumors near structures at risk.^14^ Nonetheless, quantitative analysis of the effects of these parameters has not been done, and their impact on the resulting ablation zone is not clear. Additionally, it is well known that the characteristics of both the target tissue (the nidus in this case) and the tissue surrounding it have a big impact on the resulting ablation zone. To explore the effects of all these parameters in a systematic way, we developed finite element models to simulate RFA of OO under multiple configurations of control temperature, ablation time, and anatomical location of the tumor.

In some of the first RFA studies with OO, Rosenthal et al concluded that little to no change was achieved in the extent of the ablation zone after approximately 3 min but that nevertheless, in clinical practice, the patients seemed to experience less recurrences when the duration of the RFA was increased to 6 min^36^ Other studies even claimed to notice a difference in the clinical outcomes by increasing the ablation time to up to 15 min.^12^ Our results showed that an ablation tends to develop rapidly, achieving most of the ablation radius during the first few minutes, and then actually grows slowly so that it develops considerably during the whole 15 min of active heating and perhaps even longer. This was true for all the simulated OO geometries and for every control temperature, and especially true for geometry (c) where the ablation radius was the largest and it grew the most with time.

A possible explanation for the different radii obtained with each model may be due to the effects of blood perfusion. Schutt and Haemmerich^37^s showed that cirrhotic livers (with lower perfusion rates) had larger ablations zones than normal healthy livers because of what has been called the “oven effect.” Highly perfused tissues usually act as heat sinks, increasing the rate in which heat is drawn away, whereas non‐perfused tissues surrounding the tumor could be seen as a sort of oven, as with cirrhotic livers, allowing more heat to concentrate in the tumor and its surroundings. Cortical bone also has little to no blood perfusion. A nidus surrounded by cortical bone could therefore be compared to cirrhotic livers, with cortical bone causing the oven effect. This could also explain why the tumor surrounded by trabecular bone had the smallest ablation radius of all, as the trabecular bone was modeled with the highest perfusion of all the three models whereas in the case of geometry (b), it was immediately next to the tumor, acting as a heat sink. Regarding geometry (a), the electrical conductivity and perfusion modeled for the sclerotic layer was somewhere between that seen in cortical bone and trabecular bone, and the results that were somewhere between the other two models. Additionally, geometry (c)'s ablation radius grew larger in the direction perpendicular to the electrode than in the parallel direction. As the ablation radius grew in size, it reached the muscle boundary in the parallel direction, and its high perfusion could have acted as a heat sink. The nidus's blood perfusion did not seem to have a significant effect on the outcome of the ablation radius. In fact, the ablation radius quickly became bigger than the nidus, which caused the nidus's blood perfusion to stop when its cells died.

However, we think that a more plausible explanation for the overall results may be due to the differences in the electrical conductivity of the tumors and their surrounding tissue. It is known that the electrical conductivity of the target tumor is crucial in defining the volume of the ablated tissue, with higher electrical conductivities allowing for more energy deposition and thus larger ablation volumes. Yet, Solazzo et al^38^ showed that the electrical conductivity of the background tissue also has a strong influence on how the energy is deposited and therefore on the thermal distribution. They varied the electrical conductivity of the target tissue and its surroundings with a phantom and a computer model and demonstrated that when the background tissue had the same electrical conductivity as the target tissue, the electrical and thermal distribution decreased smoothly from the electrode towards the outer boundaries of the model. However, when the electrical conductivity of the background tissue was lower, they observed a secondary electric field peak at the tissue interface, which was associated with a “wider” temperature distribution (i.e., higher temperatures going deeper). When the electrical parameters were reversed, whereby the target tissue had low electrical conductivity and the surrounding tissue had increased electrical conductivity, the opposite effect occurred. In this case, there was no heat generation at the tissue interface, causing a “compressed” temperature distribution, since high temperatures were only found close to the electrode. These results are analogous to what we found where the cortical bone seemed to produce larger ablation radii because of an additional resistive heating peak at the nidus‐bone interface. This effect is more noticeable when we compare geometries (b) and (c), because trabecular bone has considerably lower electrical conductivity than cortical bone, and as such the model with trabecular bone had a smaller resistive heating peak and a smaller ablation radius.

Irastorza et al mentioned a similar trend in their computer models of OO, where they studied the presence of the layer of sclerosis using various degrees of vascularization and bone density. In their study, the ablation radius increased with decreasing electrical conductivity of the reactive zone, and an additional peak in the temperature distribution, which is similar to what Solazzo et al found.^15^, ^38^ This confirms the importance of the background tissue on the resulting ablation zone, and shows how surrounding tissue with lower conductivity can lead to an increased ablation zone. This also illustrates that while bone is a good thermal and electrical insulator, bony background tissue will alter the heat induction profile, particularly when there is a high mismatch between the target tissue and the background tissue, as shown by Solazzo et al.^38^ The consequences of this effect may seem counter‐intuitive at first, given that bone is a good insulator, but experiments have demonstrated that, particularly in the case of background cortical bone, it could potentially lead to larger ablations in comparison to other background tissues.

The effects of this mismatch in electrical conductivity were stronger when the electrical conductivity of the nidus was increased further, resulting in even larger ablation radii than the ones produced with the original settings. These variations in tumor properties, however, also showed that the blood perfusion of the tumor had little impact on the heat distribution in comparison to the electrical conductivity. This lack of dependency on the blood perfusion seemed to be because of how quick the ablation zone encompassed the nidus, stopping the nidus's blood perfusion in the first seconds to a minute of the procedure. This insight could be particularly important in cases where RFA is used to treat a malignant bone tumor because tumor tissues, especially malignant ones, seem to demonstrate much higher electrical conductivity compared to healthy tissue.

Our experiments show that ablation outcomes are highly tissue dependent, and not only on the properties of the target, but also on its surrounding tissues, which suggests the need for accurate patient specific imaging and treatment planning. This could be particularly important for tumors close to structures at risk, and considerations should be made based upon both the tumor properties and its surrounding tissue. Computational modeling for patient specific planning could play an important role to guarantee the success of the procedures.

A limitation of this study is the lack of validation of the model against clinical or in‐vivo models; it could only be validated against ex‐vivo OO experiments. However, since the model correlated accurately to the ex‐vivo experiments, one can assume that the model demonstrates the effects and importance of the tested parameters with clinically useful levels of agreement.

## 5 CONCLUSION

The ablation radius of RFA on OO is clearly dependent on the target temperature and ablation time, where higher temperatures and longer ablation times produce higher ablation radii. The ablation radii grow rapidly during the first few minutes and continue to grow, although slowly, until the electrode is turned off after 15 min. The size of the ablation radius clearly depends on the background tissue surrounding the tumor, where tissues with lower electrical conductivity led to an increased ablation radius, and this effect increases when the electrical conductivity of the tumor is increased as well. The blood perfusion of the tumor does not seem to have an important effect on the resulting ablation radius. This suggests the need for patient specific imaging and planning of the procedures, where computational models like the one presented here could help to give more accurate and personalized patient treatment.
